# Case Report: *Mycoplasma pneumoniae*-induced rash and mucositis (MIRM) in an 8-year-old girl: concise review of a challenging case.

**DOI:** 10.3389/fped.2025.1697245

**Published:** 2025-11-13

**Authors:** Yun-Long Li, He-Wen Wang

**Affiliations:** Department of Pediatrics, Rizhao People's Hospital, Shandong, China

**Keywords:** *mycoplasma pneumoniae*, rash, mucositis, child, diagosis

## Abstract

We report an 8-year-old girl presenting with an 8-day history of cough, 5-day fever, and recent-onset conjunctival hyperemia and oral mucosal erosions. Laboratory testing confirmed *Mycoplasma pneumoniae* infection (IgM antibody and DNA positive). Chest x-ray demonstrated bilateral increased and blurred lung markings, consistent with acute bronchitis. Initial treatment with azithromycin and methylprednisolone sodium succinate (MPSS) improved respiratory symptoms but triggered a progressive rash involving the trunk, perineum, and anus. Adjuvant therapy with intravenous immunoglobulin (IVIG, 22.5 g) and continued immunomodulation with MPSS led to resolution of mucocutaneous manifestations by hospital day 10. This case highlights the potential for *M. pneumoniae* to induce systemic immune-mediated reactions and underscores the importance of multimodal therapy.

## Introduction

*Mycoplasma pneumoniae* is a common pathogen responsible for community-acquired pneumonia in children, often manifesting with respiratory symptoms (1). Extrapulmonary manifestations, including dermatological involvement, are increasingly recognized. *Mycoplasma pneumoniae*-induced rash and mucositis (MIRM) is a distinct clinical entity characterized by prominent mucositis (oral, ocular, genital) with variable cutaneous involvement, often mimicking but distinct from Stevens-Johnson syndrome (SJS) and toxic epidermal necrolysis (TEN) (2). MIRM typically presents with less severe skin necrosis and a stronger association with M. pneumoniae infection compared to SJS/TEN (3). Diagnosis relies on recognizing the clinical pattern, confirming M. pneumoniae infection, and excluding other severe drug reactions. This case report describes the presentation, diagnostic journey, and successful management of MIRM in a child.

## Case report

**Figure 1:**
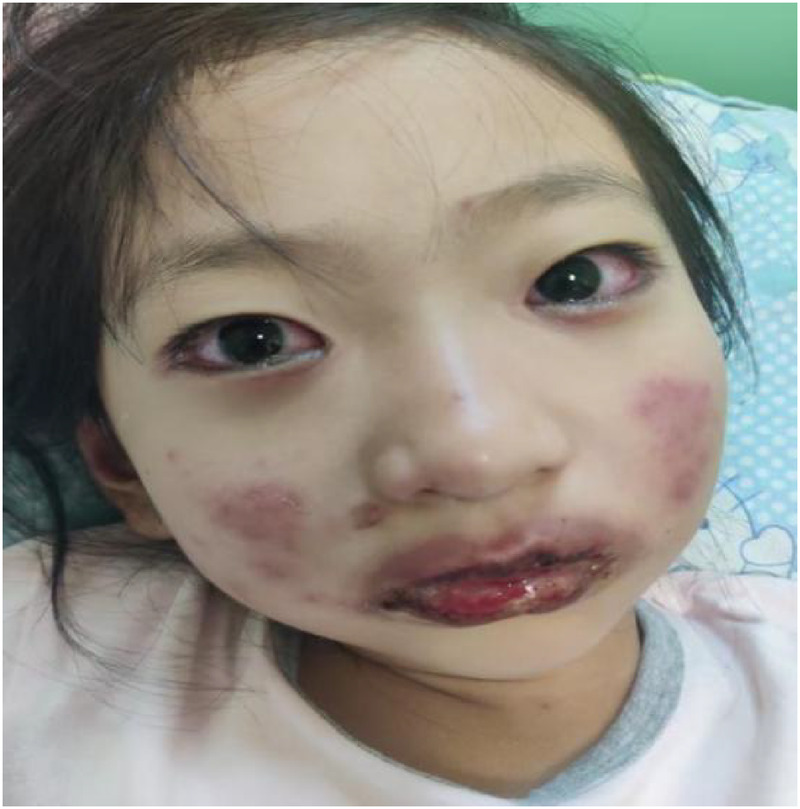
Mouth and Eye.

An 8-year-old girl presented to the pediatric ward with an 8-day history of cough, 5 days of fever, and 2 days of bilateral conjunctival congestion and painful lip erosions (Figure 1). Physical examination confirmed conjunctival hyperemia and significant erosions of the lips. Initial laboratory investigations confirmed acute *Mycoplasma pneumoniae* infection (positive serum IgM antibodies and PCR detection of M. pneumoniae DNA). Chest radiography revealed bilateral increased and blurred lung markings, leading to a diagnosis of acute bronchitis.

**Figure 2:**
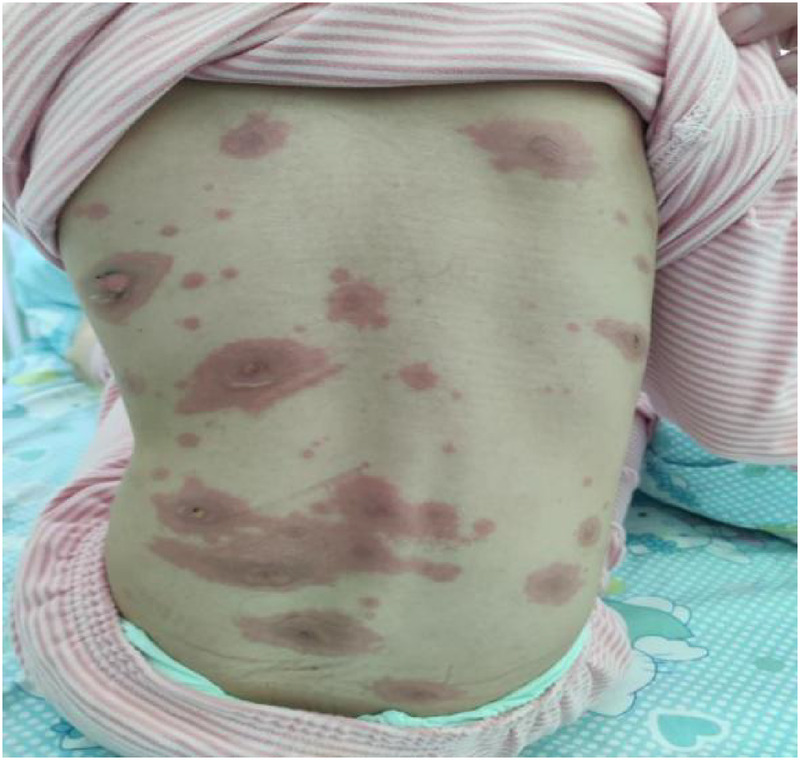
Trunk.

**Figure 3:**
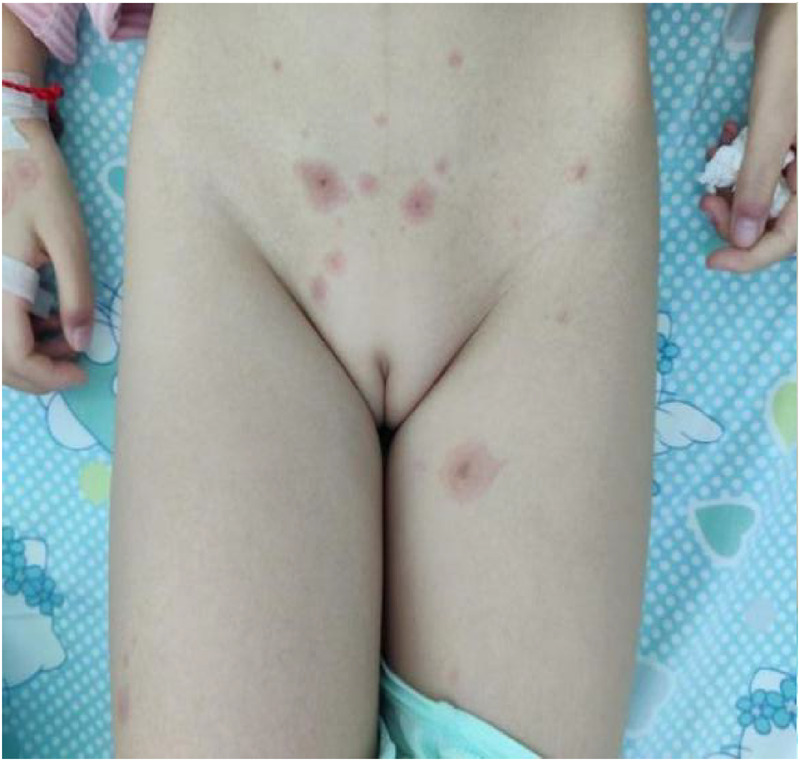
Perineum.

**Figure 4:**
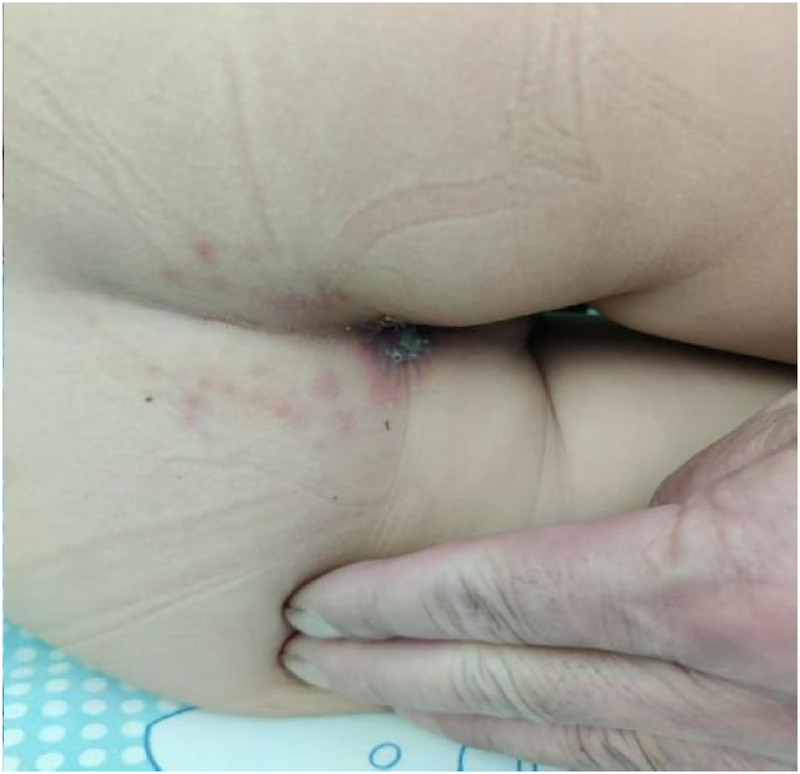
Anus.

Treatment was initiated with intravenous azithromycin (10 mg/kg/day) and intravenous methylprednisolone sodium succinate (MPSS, 2 mg/kg/day). While this regimen resulted in improvement of respiratory symptoms (cough and fever), the mucocutaneous condition deteriorated unexpectedly. A progressive maculopapular rash developed on the trunk (Figure 2), extending to involve the perineum (Figure 3) and perianal region (Figure 4). The lip erosions worsened, and ocular symptoms persisted.

Given the confirmed M. pneumoniae infection and the evolving clinical picture characterized by severe mucositis (oral, ocular, genital) with a widespread but non-necrotic rash, the diagnosis was revised to *Mycoplasma pneumoniae*-induced Rash and Mucositis (MIRM). Based on the skin and mucosal manifestations, MIRM is classified into three types: the classic type (few bullous eruptions or atypical target lesions), the non-bullous type, and the severe type (generalized disseminated bullous eruptions or atypical target lesions) (4). This patient presented with numerous atypical target lesions and bullous eruptions, which was classified as severe-type MIRM. This diagnosis was made after careful differentiation from SJS and TEN, which typically present with more extensive epidermal detachment and necrosis, and are often more strongly linked to drug reactions.

Subsequently, the therapeutic management was intensified with a multi-pronged approach. Immunomodulation was achieved through intravenous immune globulin (IVIG) administered at a total dose of 22.5 g (approximately 1 g/kg) over one day, while intravenous methylprednisolone (MPSS) was maintained at 2 mg/kg/day. No additional dose of IVIG was administered due to the child's rapid clinical improvement. Concurrently, topical mucosal repair agents were implemented, utilizing Kangfuxin Solution for oral lesions and Beifuxin Gel for genital areas to promote healing. Ocular care involved the administration of tobramycin and fluorometholone eye drops to manage conjunctival inflammation and prevent secondary infection. Furthermore, comprehensive supportive care was provided, including adequate hydration, nutritional support, and systematic analgesic management.

The response to this combined therapy was favorable. The conjunctival hyperemia resolved, the mucositis significantly improved, and the widespread rash gradually subsided. By hospital day 10, the mucocutaneous lesions showed substantial healing, and the patient was discharged in stable condition with outpatient follow-up arranged. Oral steroids were tapered over the subsequent weeks.

## Discussion

MIRM was historically often categorized under Stevens-Johnson syndrome (SJS). However, due to significant differences in their treatment and prognosis, Canavan et al. proposed the concept of MIRM in 2015 (5). MIRM is caused by immunoglobulin production from B-cell clones, leading to the deposition of immune complexes in the skin and promoting activation of the complement cascade. This mechanism differs from that of SJS and toxic epidermal necrolysis (TEN), which are type IV hypersensitivity reactions mediated by Fas ligand cytotoxicity (5).

Reactive infectious mucocutaneous eruption (RIME) is a condition characterized by mucositis and skin lesions of varying severity triggered by various infections. Oral involvement is universally present, including hemorrhagic crusting of the lips and erosions of the tongue and buccal mucosa (5, 6). Most patients also have ocular and urogenital involvement. Literature reports indicate that various respiratory pathogens can lead to RIME, including *Mycoplasma pneumoniae*, Chlamydia, influenza B virus, adenovirus, etc. (7–11). The pathogenesis of RIME remains unclear and is thought to involve an immune response triggered by a distant infection, leading to damage of mucocutaneous tissues.

This case illustrates the classic biphasic presentation of MIRM: an initial respiratory phase followed by a mucocutaneous phase. The diagnosis of MIRM hinges on recognizing this pattern, confirming M. pneumoniae infection, and demonstrating the characteristic features: prominent mucositis affecting at least two sites (oral, ocular, urogenital) and a sparse, often polymorphic (maculopapular, targetoid, vesicular), non-necrotic rash, typically involving the trunk and extremities, with frequent genital involvement (2, 12).

Our patient exhibited severe oral and ocular mucositis, perineal/perianal involvement, and a widespread truncal rash, aligning perfectly with MIRM criteria. The timing of the rash after the initiation of antibiotics and steroids initially raised suspicion of a drug reaction. However, the strong evidence of M. pneumoniae infection, the specific mucocutaneous pattern (less epidermal detachment than typical SJS/TEN), and the excellent response to immunomodulation strongly supported MIRM over SJS/TEN (13). Currently, there are no treatment guidelines for MIRM. Therapeutic approaches include anti-*Mycoplasma pneumoniae* therapy (macrolides, tetracyclines, or quinolones), anti-inflammatory treatment (such as corticosteroids, intravenous immunoglobulin, etc.), and symptomatic supportive care (pain management, intravenous fluid replacement, and mucosal care, etc.) (14).

While macrolides like azithromycin are the mainstay for treating the underlying M. pneumoniae infection, the immune-mediated mucocutaneous manifestations of MIRM often require systemic immunomodulatory therapy (6). Corticosteroids are frequently used first-line, as in this case. However, the progression of mucocutaneous symptoms despite steroid initiation prompted the addition of IVIG, which is increasingly recognized as beneficial in moderate to severe MIRM, potentially halting the immune response more effectively (4, 15). The synergistic effect of continued steroids and IVIG, combined with meticulous supportive care (including targeted mucosal healing agents and ocular therapy), led to a rapid and favorable outcome in this patient. There have also been reports showing that MIRM patients with severe mucosal involvement have responded to other immunomodulatory therapies, including cyclosporine (16), intravenous immunoglobulin (IVIG), and anti-tumor necrosis factor (TNF) agents (17, 18).Therefore, we believe that immunoglobulin therapy is necessary for patients who show a poor response to early corticosteroid treatment or for those with severe MIRM.

This case underscores the importance of considering MIRM in children with M. pneumoniae infection who develop significant mucositis and rash, even after starting antimicrobial therapy. Since there are no formal guidelines, this strategy is based on established management principles for this specific condition. The key is to simultaneously address the underlying infectious trigger, control the exaggerated immune response, and provide intensive supportive care.

## Data Availability

The datasets presented in this article are not readily available because of ethical restrictions and patient privacy concerns. Requests to access the datasets should be directed to the corresponding author.
